# Neurocysticercosis presenting as hydrocephalus and bilateral optic atrophy

**DOI:** 10.4103/1817-1745.66670

**Published:** 2010

**Authors:** Rashna Dass, Himesh Barman, Saurabh Sarma, Pubali Deka, Saurabh Gohain Duwarah, Nayan Mani Deka

**Affiliations:** North Eastern Indira Gandhi Regional Institute of Health and Medical Sciences (NEIGRIHMS), Meghalaya, India; 1 Bansara Eye Care Centre, Shillong, Meghalaya, India

Sir,

Cysticercosis, the infection caused by the larval stage of the tapeworm *Taenia solium*, is the most common parasitic infestation of the central nervous system (CNS). Seizure is the most common presentation and is the single most common cause of acquired epileptic seizure in the developing world.[1] However, the clinical presentations can be pleomorphic depending on the stage and the location of the cysts in the nervous system. Neurocysticercosis (NCC) must therefore be considered in the differential diagnosis of a wide variety of neurologic disorders, particularly in endemic areas.[2] We report a case of multiple NCC with hydrocephalus with bilateral optic atrophy because of the rarity of the presentation. The relevant literature and possible pathogenesis has been reviewed.

An 8-year-old child presented with a history of diminished vision for the last 3 years. There was a history of generalized tonic–clonic seizure for the same duration. There was no history suggestive of raised intracranial pressure (ICP). On examination, visual acuity showed perception of hand movement close to the face. Fundus examination showed bilateral optic nerve atrophy. Higher mental functions and cranial nerves were intact. Tone was increased in all four limbs and there were exaggerated knee jerks. Planter showed withdrawal response on both sides. Examinations of the other systems were normal.

Magnetic resonance imaging (MRI) of the brain showed multiple NCC in vesicular stages in the bilateral cerebral parenchyma and cerebellum with no perilesional edema and mass effect. There was significant hydrocephalus due to a lesion obstructing the out flow of the fourth ventricle at the foramen of Magendi. Hemogram showed normal values and Mantoux test was negative. The child was put on phenytoin, acetazolamide and prednisolone. A right ventriculoperitoneal shunt was performed. Postoperatively, the child was stable and regained a visual acuity of finger counting at 1½ feet distance. The child was discharged at 1 week on oral prednisolone and phenytoin.

*Taenia solium*, the pork tape worm, is a two-host zoonotic cestode. Man harbors the adult tape worm in the small intestine and is the only known definitive host. As in all cestodes, the gravid proglottids at the terminal end of the worm are full of eggs, which are the source of infection with the larval stage, or cysticercosis. The pig is the natural intermediate host, which harbors larval cysts anywhere in its body. Humans become infected with cysts by accidental ingestion of *T. solium*-infective eggs by feco-oral contamination. In such a case, it preferentially infests the CNS.[1–3]

Symptomatic disease occurs almost exclusively from invasion of the CNS and the eye. NCC can be divided as parenchymal and extra-parenchymal. Seizure is the most common presentation of the parenchymal form, which accounts for 70–90% of the overall presenting features.[3] Reports of uncommon presentations of NCC are few but depict the pleomorphic nature of the disease and hence NCC should be suspected in a wide variety of neurological diseases in an endemic area.[3,4]

Visual loss in NCC is a serious consequence and can result either from direct ocular involvement or secondary to CNS involvement. It can affect any part of the visual pathway from the eye ball, optic nerve to the visual cortex. Optic nerve affection in NCC can be secondary to compression of the optic nerve in the optic canal and optic chiasma or due to a variable duration of raised ICT. Papilliedema, unilateral optic atrophy and bilateral optic atrophy have all been described. In the series of 23 cases with visual impairment reported by Chang *et al*., 13 cases were found to be due to optic neuropathy secondary to papilledema.[5] Proaño *et al*., in their series of giant neurocyticercosis, encountered a case of bilateral optic atrophy due to nerve entrapment secondary to basal arachnoididtis.[6] Similarly, Pansey *et al*. reported a 5-year-old child presenting with headache and blindness, and bilateral optic atrophy who recovered completely after antiparasitic treatment.[7] In our case, the child presented with diminished vision due to bilateral optic atrophy resulting from hydrocephalus caused by NCC. The cases reported have demonstrated a good visual recovery after VP shunt and antiparasitic treatment in their respective case reports. Our case also showed a trend toward adequate recovery following surgical management.

Hydrocephalus is a recognized presentation of extraparenchymal lesions, which occurs consequent to mechanical obstruction of the ventricles or the basal cisterns, either by the cysts themselves or by an inflammatory reaction (ependymitis and/or arachnoiditis). Because the cyst membrane is thin and the fluid is isodense with the cerebrospinal fluid, uninflamed extraparenchymal cysticerci are usually not visible on computed tomography scanning and may only reveal subtle, indirect findings on MRI. Therefore, neuroimaging may reveal hydrocephalus without noticeable cysts.[2]

The association of hydrocephalus and bilateral optic atrophy is not unique to NCC. Pojda-Wilczek studied 15 severely visually impaired children with hydrocephalus and associated diseases. They concluded that optic atrophy is the main cause of low vision in children with hydrocephalus.[8] The pathogenesis of optic atrophy associated with hydrocephalus in children could be either secondary to a long-standing unaddressed papilledema or due to stretching of the optic nerve resultant to the expanding skull.[4] The former mechanism is more likely in older children as in our case.

We report this case as a rare presentation of NCC. In our case, there were multiple intraparenchymal NCC and diagnosis was relatively easy following MR study. However, as discussed above, extraparenchymal NCC may be missed in neuroimaging. Therefore, in a child with unexplained hydrocephalus with or without bilateral optic atrophy, NCC should be considered in the differential diagnosis.
